# A novel *LARP7* variant expands the phenotypic spectrum of Alazami syndrome and reveals transcriptomic dysregulation of developmental and extracellular matrix pathways

**DOI:** 10.3389/fcell.2026.1904805

**Published:** 2026-10-14

**Authors:** Mozan Hassan, Manal Irshaid, Aalia Batool, Wadima Aldhaheri, Latifa Ba Qehaizel, Shamma Alzaabi, Shamma Alnuaimi, Sara Saeed, Fatma Al Jasmi, Nadia Akawi

**Affiliations:** Department of Genetics and Genomics, College of Medicine and Health Sciences, United Arab Emirates University, Al Ain, United Arab Emirates

**Keywords:** Alazami syndrome, dermal fibroblast, *LARP7*, RNA sequencing, tetralogy of fallot, transcriptomic dysregulation

## Abstract

**Background:**

*LARP7* encodes a critical scaffold protein of the 7SK small nuclear ribonucleoprotein (7SK snRNP) complex that regulates RNA polymerase II transcriptional elongation. Biallelic loss-of-function variants in *LARP7* cause Alazami syndrome, a rare neurodevelopmental disorder characterized by intellectual disability, growth restriction, and distinctive craniofacial features. However, the transcriptomic consequences of *LARP7* deficiency in human cells remain poorly understood, and the phenotypic spectrum of the disorder is incompletely defined.

**Methods:**

We investigated a 6-year-old boy with clinically diagnosed Alazami syndrome who carried a novel homozygous *LARP7* frameshift variant. Clinical evaluation and exome sequencing of blood-derived genomic DNA were performed, while quantitative PCR, Western blotting, and transcriptomic analysis were conducted using patient-derived dermal fibroblasts. Transcriptomic consequences of *LARP7* deficiency were assessed by RNA sequencing, followed by single-subject differential expression, pathway enrichment, and network analyses.

**Results:**

The patient presented with classical features of Alazami syndrome and tetralogy of Fallot, a cardiac manifestation not previously reported in the disorder. The novel homozygous *LARP7* frameshift variant resulted in marked depletion of *LARP7* transcripts and complete loss of full-length protein. Transcriptome-wide analysis identified 1,224 dysregulated genes involved in extracellular matrix organization, developmental remodeling, cell-cycle regulation, and immune signaling. Pathway and network analyses highlighted dysregulation of STAT3-associated signaling together with SMAD3-linked extracellular matrix and developmental programs, identifying candidate developmental and tissue-remodeling pathways that warrant further investigation in disease-relevant models.

**Conclusion:**

This study provides the first comprehensive transcriptomic characterization of genetically confirmed *LARP7* deficiency in primary human cells and potentially expands the phenotypic spectrum of Alazami syndrome. The findings reveal widespread dysregulation of developmental and extracellular matrix pathways, offering new insight into the molecular basis of the multisystem manifestations of Alazami syndrome and providing a framework for future functional and genotype–phenotype studies.

## Graphical Abstract

**Figure:**
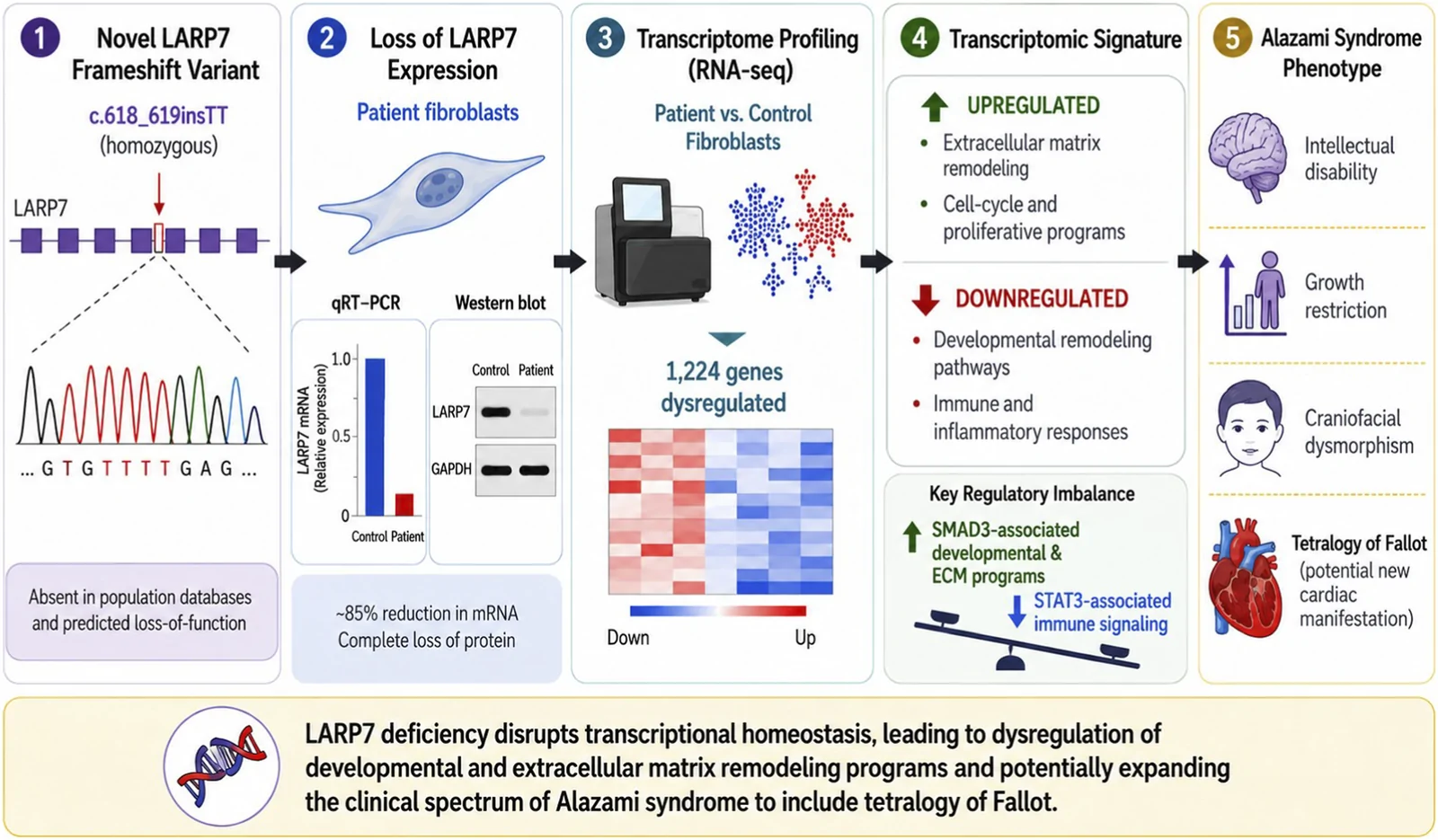

## 1 Introduction

Transcriptional pause-release by RNA polymerase II is a critical regulatory step in gene expression and is essential for normal development and cellular homeostasis. Central to this process is the positive transcription elongation factor b (P-TEFb; CDK9/Cyclin T1), whose activity is tightly controlled by the 7SK small nuclear ribonucleoprotein (7SK snRNP) complex (Dollinger and Gilmour, 2021; Egloff, 2021; Lysakovskaia et al., 2025; Naganuma et al., 2025). Within this complex, the La-related protein 7 (*LARP7*) binds and stabilizes the 7SK non-coding RNA, thereby maintaining sequestration of P-TEFb and regulating transcriptional elongation (Figures 1A–C) (Quaresma et al., 2016). Structural and functional studies have shown that disruption of the 7SK snRNP complex alters transcriptional pause-release dynamics and leads to widespread dysregulation of gene expression (Hasler et al., 2021). In addition to its role in transcriptional control, *LARP7* contributes to RNA processing and genome stability, including regulating U6 spliceosomal RNA modification and participating in DNA damage response pathways (Hasler et al., 2020; Wang et al., 2020). Together, these findings highlight *LARP7* as a multifunctional regulator of cellular integrity and developmental gene-expression programs.

**FIGURE 1:**
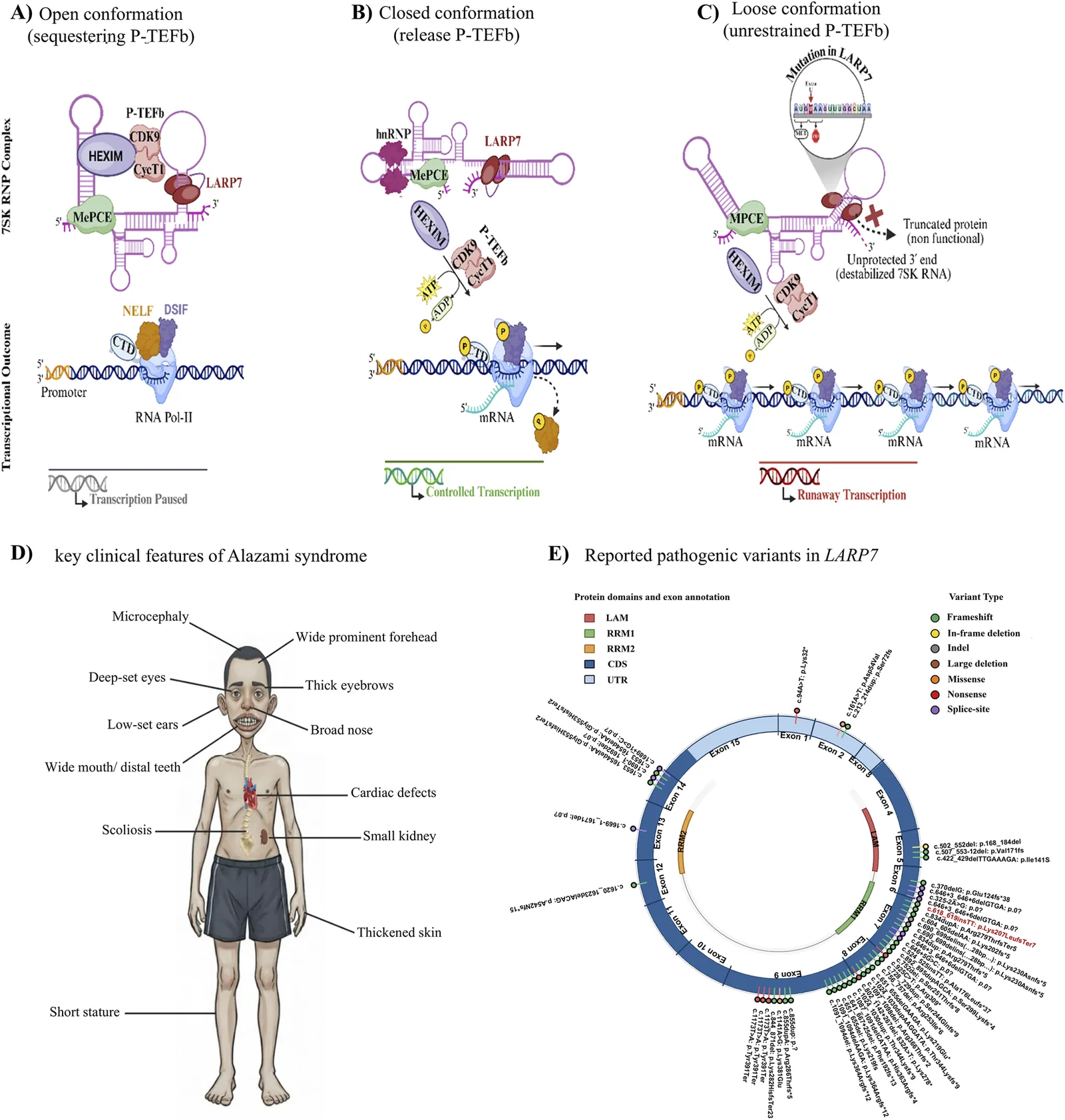
Model of 7SK snRNP regulation and the molecular and clinical consequences of *LARP7* loss. **(A)** In the open conformation, 7SK snRNP sequesters P-TEFb to maintain RNA Pol II pausing. **(B)** Upon stimulation, the complex adopts a closed conformation, releasing P-TEFb to enable controlled transcriptional elongation. **(C)** *LARP7* mutation destabilizes 7SK RNA, producing a loose conformation with unrestrained P-TEFb and runaway transcription. **(D)** Summary illustration of key clinical features of Alazami syndrome resulting from *LARP7* dysfunction. **(E)** Schematic of the *LARP7* gene showing protein domains, exon organization, and reported pathogenic variants. The frameshift variant identified in this study (c.618_619insTT; p.(Lys207LeufsTer7)) is shown in red.

Biallelic pathogenic variants in *LARP7* cause Alazami syndrome (OMIM 615071), a rare autosomal recessive neurodevelopmental disorder characterized by severe intellectual disability, proportionate short stature, and distinctive craniofacial dysmorphism (Figure 1D; Table 1). Most pathogenic variants identified to date are predicted loss-of-function alleles (Figure 1E), consistent with impaired stability or function of the LARP7 protein. Although the clinical spectrum of Alazami syndrome has gradually expanded, the molecular mechanisms linking *LARP7* deficiency to multisystem developmental abnormalities remain poorly understood. Furthermore, congenital heart disease has only rarely been described, and tetralogy of Fallot (TOF) has not previously been reported in association with the disorder.

**TABLE 1:** Reported *LARP7* variants and associated clinical phenotypes in individuals with Alazami syndrome.

#	Identified *LARP7* variant	Gender	Age	Low body weight	Short stature	Microcephaly	Broad nose	Deep seat eyes	Strabismus	Abnormal philtrum	Triangular face	Low set ears
1	c.1024_1030dupAAGGATA	M	12	+	+	+	+	+	-	+	+	+
2	c.1024_1030dupAAGGATA	F	5	+	+	+	+	+	+	+	+	+
3	c.1024_1030dupAAGGATA	M	22	+	+	+	+	+	+	+	+	-
4	c.1024_1030dupAAGGATA	M	20	+	+	+	+	+	+	+	+	+
5	c.1024_1030dupAAGGATA	M	15	+	+	+	+	+	-	+	+	+
6	c.1024_1030dupAAGGATA	F	17	+	+	+	+	+	-	+	+	-
7	c.1024_1030dupAAGGATA	M	10	+	+	+	+	+	+	+	+	+
8	c.1024_1030dupAAGGATA	F	8	+	+	+	+	+	-	+	+	-
9	c.1024_1030dupAAGGATA	F	5	+	+	+	+	+	-	+	+	+
10	c.1091_1094delAAGA	M	3.5	+	+	+	+	-	-	+	+	+
11	c.370delG c.641_667 + 25del	M	2	+	+	+	+	+	-	-	_	-
12	c.524_525insTT	F	26	+	+	+	+	+	-	+	-	-
13	c.524_525insTT	F	22	+	+	+	+	-	-	-	-	-
14	c.161A>T	F	15	NA	NA	-	+	-	-	+	+	-
15	c.651_655delGAAGA	F	9	NA	NA	-	+	-	-	+	+	-
16	c.651_655delGAAGA	M	13	NA	NA	-	+	+	-	+	-	+
17	c.1097_1098del	M	10	NA	NA	+	-	-	-	-	-	-
18	c.728_729dup	M	20 months	+	+	+	-	+	-	-	+	-
19	c.1653_1654delAA	F	19	+	+	+	+	-	+	+	-	+
20	c.1653_1654delAA	M	29	NA	NA	NA	+	-	-	+	-	-
21	c.646 + 3_646+6delGTGA	M	6	+	+	+	+	+	+	-	+	+
22	c.1173T>A	F	5	+	+	+	+	+	+	+	-	-
23	c.646 + 3_646 + 6 delGTGA	M	5	+	+	+	+	-	-	-	-	-
24	c.1173T>A c.1653_1654delAA	F	20	+	+	+	+	+	+	+	+	+
25	c.1173T>A c.1653_1654delAA	M	12	+	+	+	+	+	+	+	+	-
26	c.646 + 3_646+6delGTGA	F	13	+	+	+	+	-	-	+	+	-
27	c.646 + 3_646+6delGTGA	M	11	+	+	NA	+	-	-	+	+	-
28	c.1173T>A	M	15	+	+	+	-	+	-	-	-	+
29	c.1173T>A	F	8	+	+	+	+	+	+	-	-	+
30	c.1173T>A	F	4	+	+	NA	+	+	-	-	-	+
31	c.892_895dupAGCA c.1087_1091delCATAA	M	19	+	+	+	-	+	-	-	+	-
32	c.855dupA	M	15	-	+	+	+	+	-	+	+	+
33	c.855dupA	M	9	-	+	+	+	+	-	+	+	+
34	c.844_871del	M	13	+	+	+	+	+	-	+	+	-
35	c.325–2A>G	F	44	NA	+	+	-	+	-	-	+	-
36	c.325–2A>G	F	42	NA	+	+	-	+	-	-	-	+
37	c.756_757del	M	8	NA	+	-	+	-	-	-	-	-
38	c.1091_1094del	M	6	+	+	+	+	+	-	+	-	+
39	c.1045_1051 dupAAGGATA	M	2.5	+	+	+	+	+	-	+	+	-
40	c.213_214dup c.651_655del	F	2	+	+	+	+	+	-	-	+	+
41	c.802_1142 + 267del	M	8 months	NA	NA	+	+	+	-	+	-	-
42	c.802_1142 + 267del	F	7 months	NA	NA	+	+	+	-	+	-	-
43	c.1689 + 1G>C	F	12	NA	+	+	+	-	+	+	-	-
44	c.1689 + 1G>C	F	6	NA	+	+	+	-	+	+	-	-
45	(c. 925 C > T)	M	15	+	+	+	+	-	-	-	-	+
46	(c.16691_1671 del)	F	25	+	+	+	+	-	-	+	-	+
47	c.16901_1692 del c.855dup	M	16	NA	NA	+	+	-	-	+	+	-
48	c.834dupA c.646 + 5G>C	M	17 months	-	-	+	+	-	-	-	-	-
49	c.502_552del	F	26	NA	NA	+	-	-	-	-	-	-
50	c.502_552del	F	21	NA	NA	+	-	-	-	-	-	-
51	c.502_552del	F	20	NA	NA	+	-	-	-	-	-	-
52	c.1620_1623delACAG	F	17	+	+	+	+	+	+	+	-	-
53	c.834dup c.690_699delinsTCCAAGCCAATAGACAATATCCAAGCC	F	17	+	+	+	+	+	+	+	-	-
54	c.690_699delinsTCCAAGCCAATAGACAATATCCAAGCC	M	17	+	+	+	+	-	-	-	-	-
55	c.690_699delinsTCCAAGCCAATAGACAATATCCAAGCC	F	9	+	+	+	+	-	-	-	-	-
56	c.604_605delAA	M	11 months	+	+	+	-	+	-	-	+	+
57	c.752del	M	6	NA	NA	-	-	-	-	-	-	-
58	c.507_553–12 del	F	10	+	+	-	-	-	-	-	-	-
59	c.422_429delTTGAAAGA	F	4	+	+	+	-	+	-	+	+	-
60	c.618_619insTT	M	6	+	+	-	+	+	+	-	+	+
Overall				40/60 (66%)	47/60 (78%)	50/60 (83%)	47/60 (78%)	36/60 (60%)	15/60 (25%)	35/60 (58%)	29/60 (48%)	23/60 (38%)

Short and/narrow palpebral fissure	Malar hypoplasia	Sparse eyebrows	Wide mouth	Spaced teeth	Intellectual disability/Development delay	Autism spectrum	Musculo- skeletal abnormalities	Thickened skin over hands/feet	Neurological abnormalities	Cardiac septal defects	Other findings	References
+	+	+	+	+	+	-	+	+	-	-	Retractile left testis	(Alazami et al., 2012)
+	+	+	+	+	+	-	+	+	-	-	-	
+	+	+	+	+	+	-	-	+	-	-	Self-mutilation of the hands	
+	+	+	+	+	+	-	-	-	-	-	-	
+	+	+	+	+	+	-	-	-	-	-	-	
-	-	+	+	-	+	-	+	-	-	-	-	
+	+	+	+	+	+	-	+	+	-	-	Self-mutilation of the hands	
-	+	+	+	+	+	-	-	-	-	-		
+	+	+	+	+	+	-	-	-	-	-	-	
+	-	-	-	-	+	-	-	-	+	-	Posterior cleft palate, micropenis	(Buisine-Sbraggia et al., 2025)
+	+	-	-	-	+	_	-	-	+	-	Severe hypotonia in infancy	(Dateki et al., 2018)
+	-	+	+	+	+	+	+	+	+	-	Cutis marmorata	(Imbert-Bouteille et al., 2019)
-	+	-	+	+	+	+	-	+	-	+	Cutis marmorata	
-	+	-	+	+	+	-	-	-	+	-	-	(Ambrose et al., 2025)
-	+	-	+	+	+	-	-	-	+	-	-	
-	-	-	+	+	+	+	+	-	+	-	ADHD, GERD, constipation, urinary incontinence, anxiety	
-	-	+	-	-	+	-	+	-	-	+	Deafness, acrocyanosis, palmoplantar hyperhidrosis	(Palumbo et al., 2020)
+	+	-	-	-	+	-	+	-	+	+	Sleep apnea, bilateral testicles in the inguinal canals, hypertelorism, telecanthus, bilateral syndactyly, oropharyngeal dysphasia, anaemia	(Fauntleroy-Love et al., 2023)
+	-	-	-	-	+	+	-	-	+	-	Hyperthyroidism	(Al-Hinai et al., 2022)
+	-	-	-	-	+	-	-	-	-	-	-	
+	+	+	-	-	+	-	-	-	-	-	Micropenis	
-	-	-	-	-	+	-	-	-	-	+	Conductive hearing loss, renal artery stenosis	
-	-	-	-	-	+	+	-	-	-	-	-	
-	-	+	+	-	+	-	-	-	+	-	Ectopic kidney	
-	+	-	+	-	+	-	-	-	+	-	-	
-	-	-	-	-	+	-	-	+	+	-	Truncal obesity with acne vulgaris	
-	-	-	-	-	+	-	-	-	-	-	Undescended testis	
-	-	-	-	-	+	-	-	-	-	+	-	
-	-	-	-	-	+	-	+	-	-	+	-	
-	-	-	-	-	+	-	-	-	-	+	-	
-	+	-	+	-	+	-	+	-	+	+	Thyroid carcinoma	(Ivanovski et al., 2020)
-	+	+	-	+	+	-	+	-	+	-	-	(Gana et al., 2020)
-	+	+	-	+	+	-	+	-	+	+		
-	-	+	+	-	+	-	+	-	-	-	Café au lait macule on right forearm, inguinal hernia	(Das et al., 2021)
-	-	-	-	-	+	-	+	-	-	-	hysterectomy	
-	-	-	-	-	+	-	-	-	-	-	hysterectomy	
-	-	-	-	-	+	-	+	-	-	+	Small kidneys, anaemia	(Holohan et al., 2016)
-	-	-	-	+	+	-	+	+	+	-	Tonsillectomy, transient erythroblastopenia	(Hollink et al., 2016)
+	+	+	+	+	+	-	+	-	-	-	-	
-	+	-	+	+	+	-	-	-	-	-	-	(Ling and Sorrentino, 2016)
-	-	-	+	-	+	+	-	-	+	-	Pulmonary stenosis, hyperkeratosis, Arnold Chiari malformation type 1	(Elmas et al., 2019)
-	-	-	+	-	+	+	-	-	+	-	Pulmonary stenosis, hyperkeratosis, corpus callosum agenesis	
-	-	+	-	-	+	-	-	-	+	-	Micrognathia	(Hosseini et al., 2019)
-	-	+	-	-	+	-	-		-	-	Seizure, micrognathia	
-	+	-	-	-	+	-	+	-	+	+		(Kazemi et al., 2020)
-	+	-	-	-	+	-	-	-	-	-	Hypercholesterolemia, bilateral cataract	
-	-	-	-	+	+	-	-	-	+	-	Phenylketonuria	(Patalan et al., 2022)
-	-	-	-	-	+	-	+	+	+	-	Aspiration, ventriculomegaly, thin corpus callosum, Micrognathia	(Wojcik et al., 2019)
-	-	-	-	-	+	-	-	-	+	-	-	(Bajafar et al., 2019)
-	-	-	-	-	+	-	-	-	-	-	-	
-	-	-	-	-	+	-	-	-	-	-	-	
+	+	+	-	+	+	-	+	-	-	-	Small left kidney, prehypertension	(Alzahrani and Bird, 2019)
-	-	-	-	-	+	-	+	-	+	-	-	(Soengas-Gonda et al., 2023)
-	-	-	+	+	+	-	+	-	+	-	-	
-	-	-	-	-	+	-	+	-	-	-	-	
-	+	+	-	-	+	-	+	-	-	+	Torticollis, undescended testis, self-mutilation hearing loss	(Thouqan et al., 2025)
-	-	-	-	-	+	-	-	-	-	-	-	(Saleh et al., 2021)
-	-	-	-	-	+	-	-	-	+	-	-	(Alsharif et al., 2022)
+	-	-	-	-	+	-	-	-	-	-	Hyperthyroidism	(Irshaid et al., 2024)
+	-	+	+	-	+	-	-	-	-	+	Inguinal hernia, TOF	This study
18/60 (30%)	24/60 (40%)	22/60 (36%)	24/60 (40%)	21/60 (35%)	60/60 (100%)	7/60 (12%)	24/60 (40%)	9/60 (15%)	25/60 (41%)	13/60 (21%)	-	

Previous studies investigating *LARP7* function have largely relied on experimental knockdown or knockout systems, providing important mechanistic insights into transcriptional regulation and cellular proliferation (Okamura et al., 2012; Holohan et al., 2016; Slomnicki et al., 2016; Hosseini et al., 2019; Hasler et al., 2020; Zhang et al., 2020). However, transcriptome-wide characterization of genetically confirmed *LARP7* deficiency in primary human cells remains limited. Defining the downstream transcriptional consequences of *LARP7* loss in patient-derived tissues may provide important insight into the developmental pathways disrupted in Alazami syndrome and help explain its phenotypic variability.

Here, we describe a 6-year-old male with clinically and molecularly confirmed Alazami syndrome caused by a novel homozygous frameshift variant in *LARP7*, who additionally presented with TOF. To investigate the molecular consequences of *LARP7* deficiency, we performed transcriptome-wide RNA sequencing of patient-derived fibroblasts. Differential gene expression and pathway analysis revealed widespread dysregulation of extracellular matrix organization, proliferative signaling, and developmental pathways. These findings potentially expand the phenotypic and molecular spectrum of Alazami syndrome and provide new insight into the transcriptional networks affected by *LARP7* deficiency.

## 2 Materials and methods

### 2.1 Patient recruitment and ethics

Members of a consanguineous Emirati family were enrolled in the study following clinical assessment of the proband at the Genetics Clinic, Tawam Hospital (Al Ain, UAE). The protocol was approved by the Department of Health, Abu Dhabi (DOH/CVDC/2023/1950), and written informed consent was obtained from all participants (parental consent was obtained for the proband).

### 2.2 Sample collection and nucleic acid extraction

Peripheral blood was drawn from the proband and available relatives into EDTA tubes; plasma was immediately separated, and genomic DNA was extracted using the QIAamp DNA Mini Kit (Qiagen) per the manufacturer’s protocol. Skin biopsies from the patient and an unrelated healthy Emirati control were cultured as dermal fibroblasts under the same experimental conditions, in DMEM supplemented with 10% fetal bovine serum (FBS) and 1% penicillin/streptomycin at 37 °C, in a humidified atmosphere containing 5% CO_2_ (Akawi et al., 2016). Both patient and control fibroblasts were expanded to passage number 4 before RNA extraction. Total RNA was extracted from fibroblasts (QIAamp RNA Mini Kit, Qiagen), and protein was prepared for Western blotting (RIPA buffer). RNA concentration and purity were checked by NanoDrop spectrophotometry (Thermo Fisher Scientific).

### 2.3 Exome sequencing

Exome sequencing was conducted at the UAEU Genomic Lab, College of Medicine and Health Sciences, UAE University, Al Ain, UAE. Genomic DNA was fragmented to an average fragment size of 180–280 bp using the LE-220 Plus Focused Ultrasonicator (Covaris, Woburn, MA, United States). Library preparation and target enrichment were performed with the TruSeq DNA Exome kit (Illumina, United States), and sequencing was conducted on the NovaSeq 6000 platform (Illumina, San Diego, CA, United States) using S1 and SP flow cells with paired-end reads. Clean reads were aligned to the human genome assembly (hg19); data processing, including read mapping, alignment, and variant calling, was carried out using a combination of the Illumina DRAGEN Bio-IT Platform (Illumina, United States) and in-house bioinformatics pipelines. Variant annotation and prioritization were conducted with VarSeq 2.2.4 (Golden Helix, United States), complemented by manual curation.

Variants passing quality control thresholds (genotype quality >30; read depth >10) were further filtered based on minor allele frequency (<0.01 in the gnomAD database) and genomic context, retaining those within coding regions and exon–intron boundaries (±20 bp), including missense, nonsense, frameshift, and splicing variants in disease-associated genes. Annotation and functional interpretation incorporated multiple resources, including gnomAD, HGMD, ClinVar, OMIM, NCBI RefSeq, dbSNP, SIFT, ExAC Gene Constraint metrics, PolyPhen-2, GERP++, MaxEntScan, and GeneSplicer. The pathogenicity of candidate variants was assessed in accordance with the American College of Medical Genetics and Genomics (ACMG) guidelines. Key TOF/CHD-associated genes, including *NOTCH1, FLT4, MYH6, NKX2-5, TBX1, JAG1, TBX5,* and *KDR*, were assessed for potentially pathogenic variants (Supplementary Table S1).

### 2.4 RNA sequencing

Stranded mRNA libraries were prepared from total RNA following the DNBSEQ protocol (MGI Tech, Shenzhen, China) (Huang et al., 2017). Briefly, poly(A)+ RNA was enriched, fragmented, and reverse transcribed to cDNA, followed by second-strand synthesis incorporating dUTP. After end repair and adaptor ligation, uracil-DNA glycosylase treatment selectively degraded the second strand to maintain directionality. DNA nanoballs were generated and sequenced on the DNBSEQ platform (PE150). Raw reads were quality-filtered and adapter-trimmed using SOAPnuke v2.3, then aligned to the human reference genome (hg38, UCSC) with HISAT2 v2.0.4 (Kim et al., 2015). Transcript-level quantification was performed using Bowtie2.

Given the absence of biological replicates, conventional replicate-based differential-expression methods were not appropriate; therefore, differential gene expression analysis was carried out using a single-subject (N-of-1) design specifically developed for non-replicated transcriptomic comparisons. This approach estimates individualized gene-level perturbations via iDEG modeling (Wang et al., 2015; Li et al., 2019), and subsequently infers pathway-level alterations using N-of-1 gene set enrichment (MixEnrich) (Berghout et al., 2017; Li et al., 2017; Aberasturi et al., 2021). The combined pipeline has been shown to maintain statistical validity in non-replicated designs and to enhance the detection of biologically meaningful pathway alterations. Recent evidence further supports gene-set-level single-subject analysis as a useful approach for recovering biologically consistent transcriptomic signatures when conventional cohort sizes are difficult to achieve (Wilson et al., 2026).

#### 2.4.1 Data preprocessing and normalization

Gene-level raw counts were imported into R (v4.5.0) and analyzed with Bioconductor packages. Genes with <10 reads across all samples were excluded (Chen et al., 2016). Counts were normalized using the trimmed mean of M-values (TMM) method implemented in edgeR (Robinson and Oshlack, 2010), and expression values were expressed as counts-per-million (CPM) and log_2_(CPM+1).

#### 2.4.2 Single-subject differential expression (iDEG)

Local variance was estimated across ranked gene windows, and patient-to-control differences were transformed into Z-scores. Empirical Bayes modeling was used to estimate local false discovery rates (locfdr). Genes were considered differentially expressed at |Z| ≥ 2 and locfdr ≤0.20.

#### 2.4.3 Pathway enrichment analysis (N-of-1 MixEnrich)

N-of-1 MixEnrich was performed using MSigDB GO: BP gene sets (v2025.1). Competitive Fisher’s exact tests were applied, effect sizes reported as odds ratios (OR), and multiple testing correction performed using the Benjamini–Yekutieli procedure. Pathways with a Benjamini–Yekutieli-adjusted FDR <0.05 were considered significantly enriched. To reduce redundancy, the top 50 enriched pathways were consolidated by semantic similarity using the GOSemSim package (Wang method) (Yu et al., 2010).

#### 2.4.4 Network-based prioritization of key driver genes

Protein–protein interaction subnetworks were built in STRING v12 (interaction confidence ≥0.9, fallback ≥0.7). Node centrality metrics (degree, betweenness) were calculated using igraph, and candidate driver genes were prioritized based on transcriptomic evidence (|Z| ≥ 2, locfdr ≤0.20), network centrality, and multi-pathway membership. Top-ranked drivers were nominated per pathway. To validate GO-based enrichment, MixEnrich was repeated using KEGG and Reactome gene sets (MSigDB C2). Gene-level concordance across databases was evaluated, and pathway overlaps were visualized using UpSet plots, Jaccard heatmaps, and Sankey diagrams.

### 2.5 Quantitative real-time PCR

Total RNA was reverse transcribed into cDNA using the GoScript™ Reverse Transcription System (Promega, United States) following the manufacturer’s instructions. Quantitative PCR was performed on an Applied Biosystems QuantStudio™ 7 Flex instrument using the TaqMan™ Gene Expression Assay for *LARP7* (Hs00277883_m1, Thermo Fisher Scientific, United States). GAPDH served as the endogenous control; each reaction was run in technical triplicate, and relative gene expression levels were calculated using the ΔΔCt method.

### 2.6 Western blotting

Total protein was extracted from fibroblast cultures using RIPA buffer supplemented with protease inhibitors. Protein concentrations were determined using the BCA Protein Assay Kit (Thermo Fisher Scientific, United States). Equal amounts of total protein were separated by SDS-PAGE and transferred to PVDF membranes. Membranes were blocked in 5% non-fat milk and incubated overnight at 4 °C with anti-LARP7 antibody (rabbit polyclonal, Proteintech) with optimized conditions. GAPDH was used as a loading control. After incubation with HRP-conjugated secondary antibodies, signals were visualized using enhanced chemiluminescence (ECL) detection (Pierce ECL Plus, Thermo Fisher Scientific, United States).

## 3 Results

### 3.1 Typical features of Alazami syndrome in the patient with tetralogy of fallot

The proband (III:4) is a 6-year-old male born to consanguineous parents from the same tribe who share Yemeni ancestry (II:3, II:4) (Figure 2A). He had four full siblings and one paternal half-sibling. Gestation was largely unremarkable, aside from diet-controlled gestational diabetes, and a normal antenatal ultrasound was obtained at 36 weeks, preceding delivery (birth weight 2.7 kg). Within 12 h of life, he developed cyanosis and hypoxemia, prompting admission to the neonatal intensive care unit. Echocardiography confirmed classical TOF with severe pulmonary stenosis at multiple levels (valvar, subvalvar, and supravalvar), a patent foramen ovale, and congenital ostial left pulmonary artery stenosis. At 4 weeks of age, a right modified Blalock–Taussig shunt was placed, and at 13 months, he underwent complete TOF repair with a transannular patch and closure of the foramen ovale. Postoperative recovery was uneventful, and oxygen saturation normalized. Review of previously reported Alazami syndrome cases in Table 1 identified only occasional congenital cardiac abnormalities, predominantly isolated septal defects or pulmonary stenosis, whereas TOF has not been reported to date.

**FIGURE 2:**
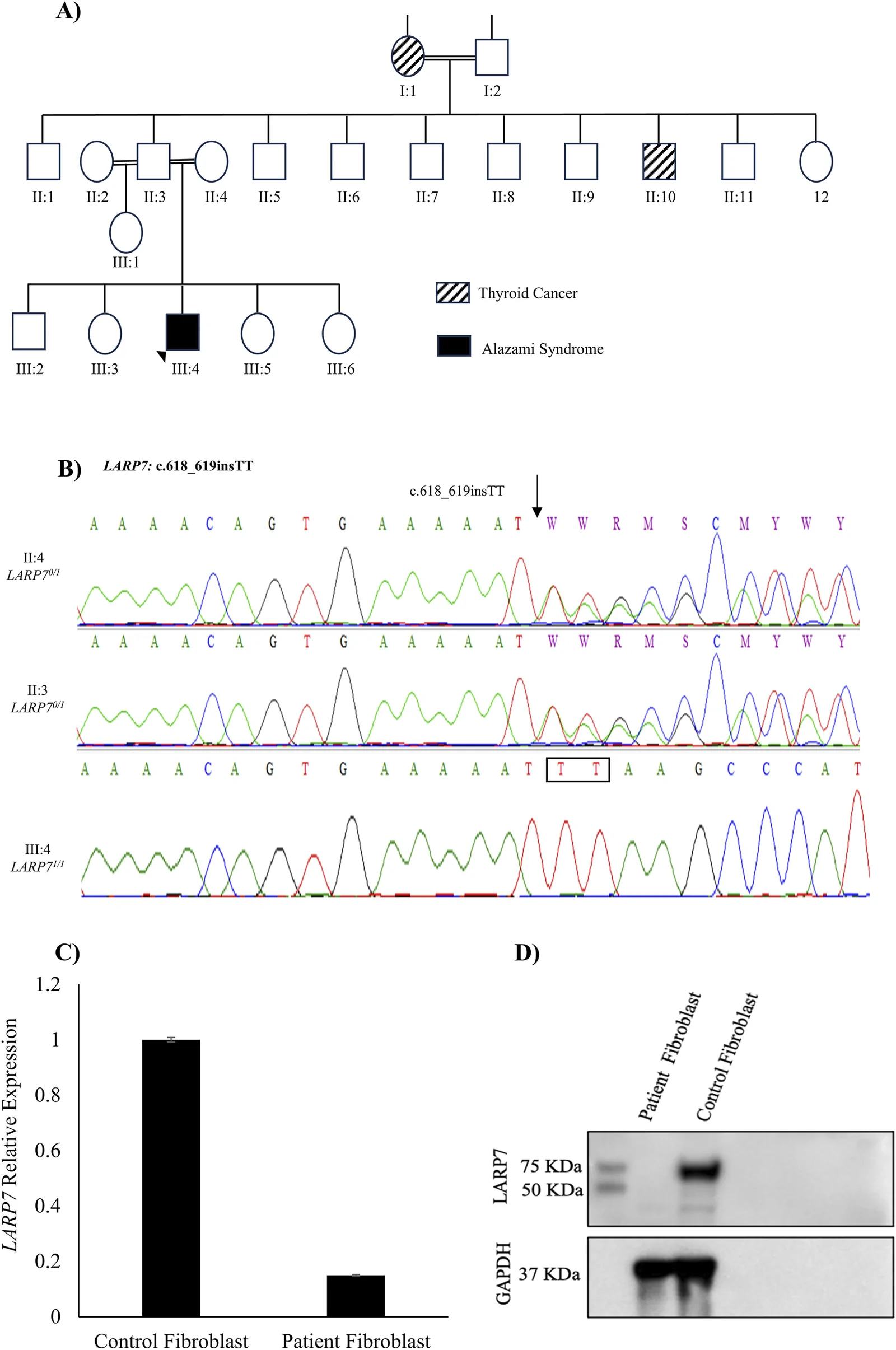
Identification and functional characterization of a pathogenic *LARP7* variant associated with Alazami syndrome. **(A)** Pedigree of the affected family showing segregation of the *LARP7* variant. Individuals with Alazami syndrome are indicated by filled symbols (black), and hatched symbols represent those with thyroid cancer. **(B)** Sanger sequencing chromatograms showing the *LARP7* c.618_619insTT insertion, the heterozygous state (II:3 and II:4) and homozygous state (III:4) for the insertion are shown. **(C)** Quantitative RT-PCR analysis of *LARP7* mRNA expression in patient fibroblasts compared to control fibroblasts. Expression is normalized to endogenous controls; data are represented as mean ± SD. **(D)** Western blot analysis of LARP7 protein levels in patient and control fibroblasts. GAPDH was used as a loading control. Reduced LARP7 protein expression is evident in patient cells.

He exhibited severe postnatal growth retardation, with height and weight persistently below the 3rd percentile. He had global developmental delay (IQ = 53) and failure to thrive, requiring prolonged nasogastric feeding. By age 5, he was not toilet trained, had a vocabulary of fewer than 10 words, and had poor comprehension; however, no autistic features were present, and his hearing was normal. He attended kindergarten (KG1) and received private speech and occupational therapy.

Distinct dysmorphic features were noted, including slanting palpebral fissures, hypertelorism, an upturned nose, and a small mouth. The patient also had microphthalmia with microcornea, large-angle esotropia, and overlapping second toes. Additional findings included surgically repaired bilateral inguinal hernias and bilateral myopia (confirmed by ophthalmologic examination). Fluorescence *in situ* hybridization (FISH) testing for 22q11.2 deletion (DiGeorge syndrome) was negative. None of the patient’s siblings showed similar developmental or cardiac anomalies, and family history was negative for related conditions, although thyroid cancer occurred in relatives II:10 and I:1 (Figure 2A).

### 3.2 DNA sequencing reveals a protein truncating variant in *LARP7*

Trio-exome sequencing identified a novel homozygous two-nucleotide insertion (TT) in *LARP7* (NM_016648.4: c.618_619insTT). This frameshift p. (Lys207LeufsTer7) alters codon 207 and introduces a premature stop codon seven residues downstream (Figure 2B). The variant was absent from the control database (gnomAD) and was unreported in clinical literature. Sanger sequencing confirmed the finding and showed complete co-segregation: both healthy parents were heterozygous carriers, consistent with autosomal recessive inheritance (Figure 2B). No other pathogenic or likely pathogenic variants were detected in genes associated with congenital heart disease or neurodevelopmental disorders. No additional pathogenic or likely pathogenic variants were identified among the assessed TOF/CHD-associated genes; detailed variant-level findings and available sequencing quality metrics are provided in Supplementary Table S1.

### 3.3 A marked reduction in *LARP7* expression was observed in patient-derived fibroblasts

Quantitative PCR demonstrated an ∼85% reduction in LARP7 transcript levels in the patient’s fibroblasts relative to a healthy control (mean ΔCt ±SD: control = 3.676 ± 0.023 vs. patient = 6.408 ± 0.033; ΔΔCt = 2.732, corresponding to a relative expression of 0.15), (Figure 2C). This pronounced transcript loss is consistent with nonsense-mediated mRNA decay triggered by the frameshift mutation. Western blot analysis corroborated these results; control fibroblast lysates showed a clear immunoreactive LARP7 band at ∼75 kDa. In contrast, the patient’s sample lacked full-length LARP7 (Figure 2D), indicating production of a truncated or unstable protein. GAPDH levels confirmed equal protein loading across samples.

### 3.4 Transcriptomic profile of patients-derived fibroblasts

RNA sequencing of patient and control fibroblasts generated ∼22 million paired-end reads per sample, with >96% Q20 and >90% Q30 base scores and >97% mapping to the hg38 reference genome (Supplementary Figure S1). A total of 12,510 genes were detected, of which 1,224 met the criteria for differential expression (|Z| ≥ 2, locfdr ≤0.20) (Supplementary Material 1). The empirical null distribution was centered near zero (Supplementary Figure S2A), and variance-stabilized transformed data revealed clear separation between the patient and control (Supplementary Figures S2B,C). The MA plot illustrates broad transcriptional shifts (Supplementary Figure S2D). Notably, several extracellular matrix (ECM) and developmental remodeling genes, including POSTN, FBN2, IGFBP3, and VCAN, were strongly upregulated, whereas genes involved in developmental signaling and differentiation, such as SFRP1, DUSP4, and DMKN, were downregulated (Figures 3A,B; Table 2; Supplementary Material 1).

**FIGURE 3:**
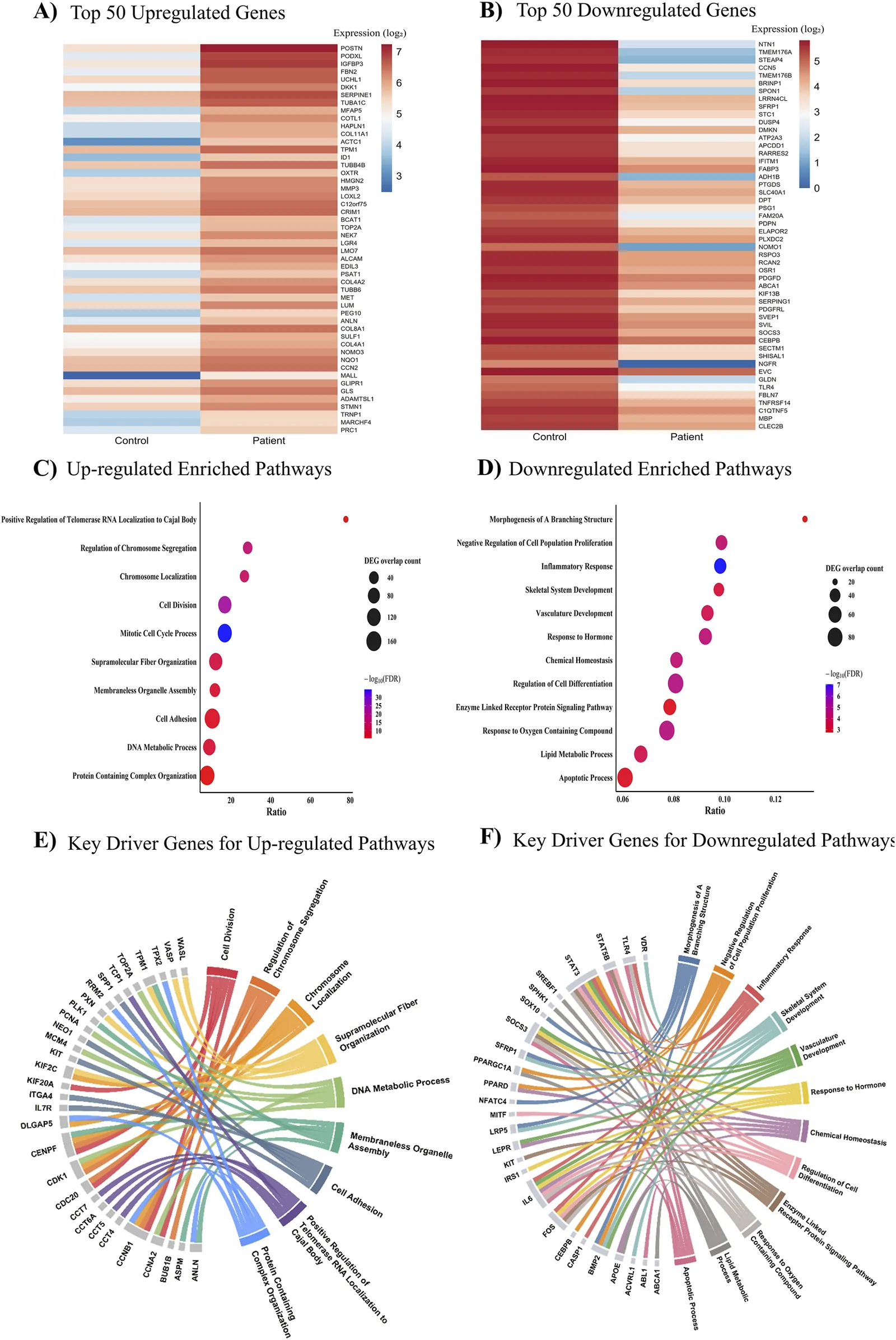
Differential gene expression and pathway analysis between patient and control samples. **(A)** Heatmap showing the top 50 upregulated genes in the patient sample compared to the control. **(B)** Heatmap displaying the top 50 downregulated genes in the patient sample. **(C)** Enriched biological pathways associated with upregulated genes, presented as a bubble plot. **(D)** Enriched biological pathways associated with downregulated genes. **(E)** Chord diagram illustrating key driver genes contributing to the upregulated pathways. **(F)** Chord diagram showing key driver genes associated with downregulated pathways.

**TABLE 2:** Top 50 upregulated and top 50 downregulated genes identified in patient-derived *LARP7*-deficient fibroblasts, ordered by Z-score.

Gene	Z-score	Direction	locfdr	Functional annotation	Gene	Z-score	Direction	locfdr	Functional annotation
POSTN	32.24	Up	0	ECM remodeling protein	NTN1	−14.65	Down	2.49E-79	Netrin-1; axon guidance
PODXL	26.72	Up	7.70E-263	Cell-surface sialomucin	TMEM176A	−13.49	Down	2.66E-66	Immunoregulatory transmembrane protein
IGFBP3	24.44	Up	1.92E-217	IGF signaling regulator	STEAP4	−12.86	Down	6.13E-59	Metalloreductase; iron/copper metabolism
FBN2	22.52	Up	7.46E-183	ECM microfibril protein	CCN5	−12.84	Down	9.48E-59	CCN5; anti-fibrotic factor
UCHL1	16.33	Up	2.17E-96	Deubiquitinase; protein turnover	TMEM176B	−12.83	Down	1.40E-58	Immunoregulatory transmembrane protein
DKK1	15.77	Up	1.43E-87	Wnt pathway inhibitor	BRINP1	−12.79	Down	2.64E-58	Neuronal tumor suppressor
SERPINE1	15.72	Up	2.03E-87	Fibrinolysis inhibitor (PAI-1)	SPON1	−11.96	Down	2.31E-50	ECM protein; axon guidance
TUBA1C	13.22	Up	3.85E-62	Alpha-tubulin; cytoskeleton	LRRN4CL	−11.02	Down	8.44E-43	LRR protein; cell adhesion
MFAP5	12.97	Up	1.21E-58	ECM glycoprotein; angiogenesis	SFRP1	−10.95	Down	1.05E-42	Soluble Wnt antagonist
COTL1	12.62	Up	3.97E-55	Actin-binding; cytoskeleton	STC1	−10.58	Down	2.45E-39	Stanniocalcin; Ca/phosphate regulation
HAPLN1	12.6	Up	4.74E-55	ECM stabilizer (hyaluronan)	DUSP4	−10.45	Down	7.83E-37	MAPK phosphatase (ERK/JNK)
COL11A1	12.53	Up	6.92E-55	Collagen; connective tissue	DMKN	−10.26	Down	3.30E-36	Dermokine; epithelial differentiation
ACTC1	11.93	Up	7.84E-49	Cardiac alpha-actin	ATP2A3	−10.23	Down	3.75E-36	ER Ca^2+^ ATPase (SERCA3)
TPM1	11.41	Up	1.03E-44	Actin-binding protein	APCDD1	−10.01	Down	1.87E-33	Wnt inhibitor; membrane glycoprotein
ID1	10.94	Up	2.56E-41	Inhibitor of differentiation	RARRES2	−9.79	Down	5.56E-33	Chemerin; chemoattractant adipokine
TUBB4B	10.77	Up	4.24E-40	Beta-tubulin; microtubules	IFITM1	−9.76	Down	6.06E-33	Interferon-induced antiviral protein
OXTR	10.76	Up	1.39E-39	Oxytocin receptor (GPCR)	FABP3	−9.5	Down	3.33E-30	Heart-type fatty-acid-binding protein
HMGN2	10.64	Up	1.84E-38	Chromatin-binding protein	ADH1B	−9.36	Down	5.38E-30	Alcohol dehydrogenase
MMP3	10.54	Up	3.33E-38	ECM protease	PTGDS	−9.01	Down	2.96E-27	Prostaglandin D2 synthase
LOXL2	10.51	Up	3.69E-38	Collagen crosslinking enzyme	SLC40A1	−8.8	Down	5.30E-25	Ferroportin; iron export
C12orf75	10.45	Up	4.56E-38	Proliferation-associated protein	DPT	−8.39	Down	1.84E-22	Dermatopontin; ECM/collagen
CRIM1	10.44	Up	4.78E-38	BMP/VEGF regulator	PSG1	−8.34	Down	2.40E-22	Pregnancy-specific glycoprotein
BCAT1	10.25	Up	1.77E-35	Branched-chain amino acid enzyme	FAM20A	−8.31	Down	2.77E-22	Pseudokinase; biomineralization
TOP2A	10.11	Up	3.67E-35	DNA topoisomerase II	PDPN	−8.26	Down	3.39E-22	Podoplanin; lymphatic marker
NEK7	10.1	Up	3.80E-35	Mitotic kinase	ELAPOR2	−8.15	Down	4.75E-22	ER/Golgi protein; autophagy
LGR4	9.76	Up	2.25E-32	Wnt/R-spondin receptor	PLXDC2	−8.12	Down	6.74E-21	Transmembrane receptor; angiogenesis
LMO7	9.76	Up	2.28E-32	Actin-associated adaptor	NOMO1	−8.09	Down	1.46E-20	Nodal modulator; embryogenesis
ALCAM	9.68	Up	3.09E-32	Cell adhesion molecule	RSPO3	−8.05	Down	2.42E-20	R-spondin-3; Wnt potentiator
EDIL3	9.66	Up	3.34E-32	Secreted ECM ligand	RCAN2	−8.01	Down	3.35E-20	Calcineurin inhibitor; NFAT signaling
PSAT1	9.53	Up	3.45E-30	Serine biosynthesis enzyme	OSR1	−7.87	Down	6.92E-20	Mesenchymal transcription factor
COL4A2	9.46	Up	7.58E-30	Collagen IV (basement membrane)	PDGFD	−7.84	Down	7.59E-20	PDGF ligand; fibroblast mitogen
TUBB6	9.34	Up	1.46E-29	Beta-tubulin isoform	ABCA1	−7.7	Down	1.88E-18	Cholesterol exporter; HDL biogenesis
MET	9.2	Up	2.26E-29	HGF receptor kinase	KIF13B	−7.65	Down	3.58E-18	Kinesin; endosome/cilium trafficking
LUM	9.07	Up	3.01E-27	ECM proteoglycan (lumican)	SERPING1	−7.59	Down	5.92E-18	C1 inhibitor; complement regulation
PEG10	9.06	Up	3.34E-27	Retrotransposon-derived protein	PDGFRL	−7.56	Down	6.69E-18	PDGFR-like decoy receptor
ANLN	9	Up	4.70E-27	Actin bundling; cytokinesis	SVEP1	−7.55	Down	7.09E-18	ECM protein; vascular/lymphatic
COL8A1	8.97	Up	5.54E-27	Collagen VIII	SVIL	−7.51	Down	8.72E-18	Supervillin; actin-myosin scaffold
SULF1	8.94	Up	6.20E-27	Heparan-sulfate endosulfatase	SOCS3	−7.37	Down	1.33E-17	JAK/STAT inhibitor
COL4A1	8.82	Up	9.21E-27	Collagen IV; basement membrane	CEBPB	−7.36	Down	1.38E-17	Transcription factor; inflammation
NOMO3	8.8	Up	9.60E-27	Nodal pathway modulator	SECTM1	−7.21	Down	5.04E-16	Immune activation ligand (CD7)
NQO1	8.79	Up	6.20E-26	Antioxidant enzyme	SHISAL1	−7.15	Down	7.30E-16	Shisa-like; Wnt/TGFβ modulation
CCN2	8.7	Up	7.90E-25	CTGF; ECM remodeling	NGFR	−6.97	Down	1.33E-15	p75 neurotrophin receptor
MALL	8.68	Up	9.60E-25	Membrane raft protein	EVC	−6.87	Down	2.33E-14	Hedgehog pathway; ciliary protein
GLIPR1	8.65	Up	1.14E-24	p53-responsive protein	GLDN	−6.86	Down	2.47E-14	Gliomedin; node of Ranvier
GLS	8.65	Up	1.17E-24	Glutaminase; metabolism	TLR4	−6.81	Down	3.84E-14	LPS sensor; innate immune activation
ADAMTSL1	8.6	Up	1.57E-24	ECM glycoprotein	FBLN7	−6.8	Down	3.89E-14	Fibulin-7 ECM protein
STMN1	8.48	Up	2.53E-24	Microtubule destabilizer	TNFRSF14	−6.75	Down	5.04E-14	HVEM receptor; T-cell signaling
TRNP1	8.46	Up	2.66E-24	Neural progenitor regulator	C1QTNF5	−6.72	Down	5.86E-14	Adiponectin-like secreted protein
MARCHF4	8.38	Up	4.90E-23	E3 ubiquitin ligase	MBP	−6.65	Down	7.41E-14	Myelin basic protein
PRC1	8.36	Up	8.35E-23	Cytokinesis regulator	CLEC2B	−6.63	Down	7.85E-14	C-type lectin; immune receptor

#### 3.4.1 Pathway enrichment analysis of differentially expressed genes

Pathway analysis using Reactome, KEGG (Supplementary Materials 2, 3), and Gene Ontology Biological Process (GO BP) yielded consistent enrichment patterns (Supplementary Figures S2E–G). Because GO BP provided the broadest and most comprehensive coverage of biologically relevant pathways, it was selected as the primary framework for downstream analysis. Using GO BP, N-of-1 MixEnrich identified 259 upregulated and 145 downregulated pathways (FDR <0.05) (Supplementary Materials 4, 5). Semantic similarity reduction grouped the top-ranked pathways into six upregulated clusters (Supplementary Material 6) and eight downregulated clusters (Supplementary Material 7). The upregulated clusters converged on proliferative processes, including mitotic cell cycle, chromosome segregation, spindle organization, and DNA metabolic processes, revealing coordinated activation of cell-cycle and genome-maintenance modules (Figure 3C).

Enrichment was also observed for pathways related to supramolecular fiber organization, cell adhesion, and tissue remodeling, consistent with the upregulation of ECM-associated genes observed among the differentially expressed transcripts. In contrast, the downregulated clusters centered on immune, homeostatic, metabolic, and developmental functions (Figure 3D), reflecting broad attenuation of tissue-regulatory signaling in LARP7-deficient fibroblasts.

#### 3.4.2 Network-based prioritization of key driver genes

Integration of transcriptomic data with protein–protein interaction networks (STRING v12) identified high-confidence driver genes within the enriched pathways (Supplementary Material 8). In the upregulated cell-cycle networks, central hub genes such as CDK1, CCNB1, CDC20, CENPF, CCNA2, TOP2A, PCNA, and KIF20A showed strong connectivity within proliferative modules (Figure 3E). Conversely, downregulated subnetworks included key immune and metabolic regulators, including STAT3, IL6, TLR4, CEBPB, and PPARGC1A (Figure 3F). Additional recurrent drivers, including IL7R, KIT, SPP1, ITGA4, and NEO1, were identified within adhesion and ECM organization networks (Supplementary Material 9). Collectively, differential expression, pathway enrichment, and network analysis converged on three major biological themes: activation of ECM and developmental remodeling programs, upregulation of proliferative and cell-cycle modules, and suppression of immune and differentiation-associated signaling pathways.

## 4 Discussion

*LARP7* enforces promoter-proximal pausing of RNA Pol II via the 7SK snRNP, ensuring precise control of developmental gene expression. In our patient, qRT-PCR showed ∼85% loss of the *LARP7* transcript, and Western blot indicated complete loss of protein function. This transcriptomic analysis of primary patient fibroblasts provides the first characterization of the molecular consequences of genetically confirmed *LARP7* deficiency in human cells. Consistent with the known role of *LARP7* in 7SK-snRNP function, marked loss of *LARP7* expression was associated with widespread transcriptional perturbation affecting multiple biological pathways.

Previous studies employing *LARP7* knockdown or knockout models have provided important mechanistic insights into transcriptional regulation and cellular homeostasis (Quaresma et al., 2016; Puidebat and Egloff, 2025). Our data complement these findings by providing a global view of the transcriptional consequences in human cells. Dermal fibroblasts retain patient-specific transcriptional signatures and are widely used in rare disease transcriptomic studies to investigate the downstream consequences of Mendelian variants (Gonorazky et al., 2019; Murdock et al., 2021; Morello et al., 2023). Accordingly, fibroblast-based RNA sequencing provides a practical framework for investigating the molecular consequences of *LARP7* deficiency in human cells.

Biallelic loss-of-function *LARP7* variants (nonsense, frameshift, splice-site) cause Alazami syndrome, characterized by short stature, developmental delay, and facial dysmorphism. The homozygous frameshift variant we identified (p.Lys207Leufs*7) fits this spectrum and explains the patient’s typical Alazami syndrome phenotype. Notably, review of published cases in Table 1 indicates that congenital cardiac abnormalities are uncommon in Alazami syndrome and have primarily consisted of septal defects or pulmonary stenosis, further emphasizing the distinctive nature of the present findings. In contrast, our patient presented with classical TOF, representing one of the most complex congenital cardiac phenotypes reported in association with *LARP7* deficiency to date. Comparison of reported *LARP7* genotypes and cardiac phenotypes (Table 1; Figure 1E) shows no clear association between cardiac involvement and a specific variant or variant class. TOF has so far been observed only in the present patient, potentially expanding the reported cardiac phenotypic spectrum of Alazami syndrome. Additional cases are needed to determine whether TOF is a recurrent feature and to establish potential genotype–phenotype correlations. The presence of TOF in our patient, together with dysregulation of developmental and ECM-related genes and pathways, highlights cardiac involvement as an area warranting further investigation in Alazami syndrome. However, the fibroblast transcriptomic findings do not establish a causal relationship with TOF. Notably, *SMAD3* expression was significantly increased in our transcriptomic dataset (Z-score = 3.5). This finding is of interest because SMAD-dependent signaling plays a central role in cardiovascular morphogenesis, including outflow tract and endocardial cushion development (Arthur and Bamforth, 2011). Experimental disruption of this pathway has been associated with conotruncal malformations, septation defects, abnormal valve formation, and abnormalities of the great arteries (Jia et al., 2007; Chakrabarti et al., 2020). Furthermore, genetic variation in *SMAD3* has been associated with congenital heart defects, including ventricular septal defects (Li et al., 2015). Notably, among the developmental genes dysregulated in our dataset, *DKK1* is expressed by cardiac neural crest cells and regulates Wnt signaling in adjacent second heart field progenitors (Wiszniak et al., 2026), while *POSTN* is expressed in developing outflow-tract cushion mesenchyme, including neural crest-derived cells (Lindsley et al., 2007). To better understand the molecular basis of these developmental abnormalities, we examined the transcriptional programs disrupted by *LARP7* deficiency.

### 4.1 Transcriptomic consequences of *LARP7* deficiency

Although loss of *LARP7* might be expected to release P-TEFb and enhance elongation, experimental evidence shows the opposite: *LARP7* depletion reduces CDK9 and Cyclin T1 levels, thereby lowering overall P-TEFb activity (Holohan et al., 2016). This compensatory downregulation has been shown to impair transcriptional elongation dynamics and may contribute to the widespread transcriptomic perturbations observed in our patient. We observed dysregulation of numerous elongation-sensitive programs, supporting the role for *LARP7* in coupling transcriptional elongation to developmental gene regulation.

ECM remodeling was a prominent signature. Several ECM-related genes (e.g., *POSTN*, *FBN2*) were markedly upregulated. *POSTN* normally functions in embryonic endocardial cushions, guiding valve, and outflow tract morphogenesis; *POSTN* knockout causes septal and outflow defects (Norris et al., 2004; Tao et al., 2011; Markwald et al., 2019), whereas *FBN2* is critical for microfibril assembly and TGF-β signaling, with mutations causing contractural arachnodactyly (Xu et al., 2020; Mead et al., 2022). Their coordinated upregulation is consistent with perturbation of developmental ECM-related programs in LARP7-deficient fibroblasts and with the enrichment of ECM organization and tissue remodeling pathways observed in our analysis. Indeed, enrichment analysis highlighted ECM organization and cell–matrix adhesion pathways. Recent developmental studies further demonstrate that tightly regulated cell–ECM interactions within the second heart field are required for cardiac outflow-tract elongation, supporting the broader relevance of ECM organization to cardiac morphogenesis (Arriagada et al., 2025). Network analysis further identified cytoskeletal regulators, including *TPM1*, *VASP*, and *PXN*, within these interconnected ECM and adhesion modules. Given the established developmental roles of these genes, these findings identify candidate pathways for further investigation.

Beyond ECM remodeling, transcriptomic analysis also revealed perturbation of proliferative and genome-maintenance pathways. Growth regulators such as *IGFBP3* and *PODXL* were upregulated, while mitotic cell-cycle and DNA replication pathways were among the most strongly enriched modules (Figure 3C; Table 2). Consistent with this pattern, central cell-cycle regulators including *CCNB1*, *CDC20*, *CDK1*, and *CENPF* occupied prominent positions within the network analysis, supporting coordinated activation of proliferative programs. These findings align with the established role of *LARP7* in transcriptional regulation, cell-cycle control, and genome maintenance (Zhang et al., 2020).

Previous studies have shown that dysregulated P-TEFb activity can impair cellular homeostasis and genome stability (Bugai et al., 2019; Fujinaga et al., 2023; Lee et al., 2024). Consistent with these observations, our analysis identified perturbation of nuclear organization pathways. *LARP7* has also been implicated in telomerase RNA stability and protection against premature senescence through SIRT1-dependent mechanisms (Yan et al., 2021). Accordingly, *LARP7* deficiency may contribute to altered nuclear architecture and cellular aging processes, potentially involving candidate driver genes identified in our network analysis, including *CCT* chaperonins and *ANLN*, consistent with prior *LARP7*-deficient aging phenotypes (Zeng et al., 2024; Cao et al., 2025).

Interestingly, thyroid cancer was reported in two relatives in the pedigree (I:1, II:10), and thyroid carcinoma has also been described in previously reported individual with Alazami syndrome (Ivanovski et al., 2020). Although a causal relationship cannot be inferred, several dysregulated genes identified in our transcriptomic analysis, including *MET* (Xu et al., 2025), *BCAT1* (Li et al., 2022), *IGFBP3* (Huang et al., 2020), *ANLN, SFRP1* (Arcinas et al., 2009), and *DUSP4* (Ma et al., 2016), have been implicated in thyroid tumorigenesis. These observations raise the possibility that altered growth-regulatory pathways may warrant further investigation in larger *LARP7*-deficient cohorts.

Whereas ECM and proliferative programs were predominantly activated, several pathways involved in differentiation, immune signaling, and tissue homeostasis were downregulated. Inhibitors of Wnt/MAPK signaling (*SFRP1*, *DUSP4*) were reduced, and cytokine signaling genes (*STAT3, STAT5B, IL6, TLR4*) were suppressed. This pattern is consistent with previous *LARP7*-deficient models that reported immune dysregulation and altered cytokine signaling (Collopy et al., 2018; Ozisin et al., 2025; Sharaf-Eldin et al., 2026). Disruption of JAK/STAT signaling likely contributes to impaired cytokine-responsive transcription, consistent with the suppression of *STAT3*, *STAT5B*, and *IL6*-associated pathways. *LARP7* also stabilizes Lin28 mRNA; its loss may push stem cells toward premature differentiation (Dai et al., 2014). Collectively, these findings suggest that *LARP7* deficiency may differentially affect multiple transcriptional programs, resulting in activation of proliferative pathways alongside suppression of developmental and immune signaling networks. This may help explain the apparent paradox of Alazami syndrome, whereby growth restriction and neurodevelopmental impairment coexist with enrichment of proliferative transcriptional programs.

### 4.2 Cardiac implications of *LARP7* deficiency

To integrate transcriptomic, pathway enrichment, and network analyses, we developed an integrated pathway model summarizing the major dysregulated biological modules identified in *LARP7*-deficient fibroblasts (Figure 4). Several dysregulated genes identified in our analysis have established roles in cardiac development (Table 3), including *ACTC1*, a congenital heart disease gene previously implicated in TOF. Among these, *STAT3* was downregulated, whereas *SMAD3* and multiple ECM-associated genes (*POSTN*, *FBN2*, *VCAN*, and *IGFBP3*) were upregulated. These findings are notable because STAT3-mediated cytokine signaling supports endocardial cushion development and cardiomyocyte survival (Wu et al., 2016; Piera-Velazquez and Jimenez, 2019), while SMAD3-associated pathways regulate ECM remodeling and morphogenetic processes required for valve and outflow tract formation (van der Pluijm et al., 2016; Hanna et al., 2021). More broadly, perturbation of cardiac developmental processes, including second heart field and outflow-tract development, has been associated with congenital heart defects, including TOF(Rochais et al., 2009; Zubrzycki et al., 2024). Together with the upregulation of cytoskeletal regulators (*TPM1*, *VASP*, and *PXN*), our findings suggest coordinated perturbation of signaling, ECM, and structural pathways involved in cardiac morphogenesis. Collectively, the observed imbalance between STAT3-associated signaling and SMAD3-associated ECM remodeling identifies transcriptional alterations associated with *LARP7* deficiency that overlap with pathways known to participate in outflow tract development.

**FIGURE 4:**
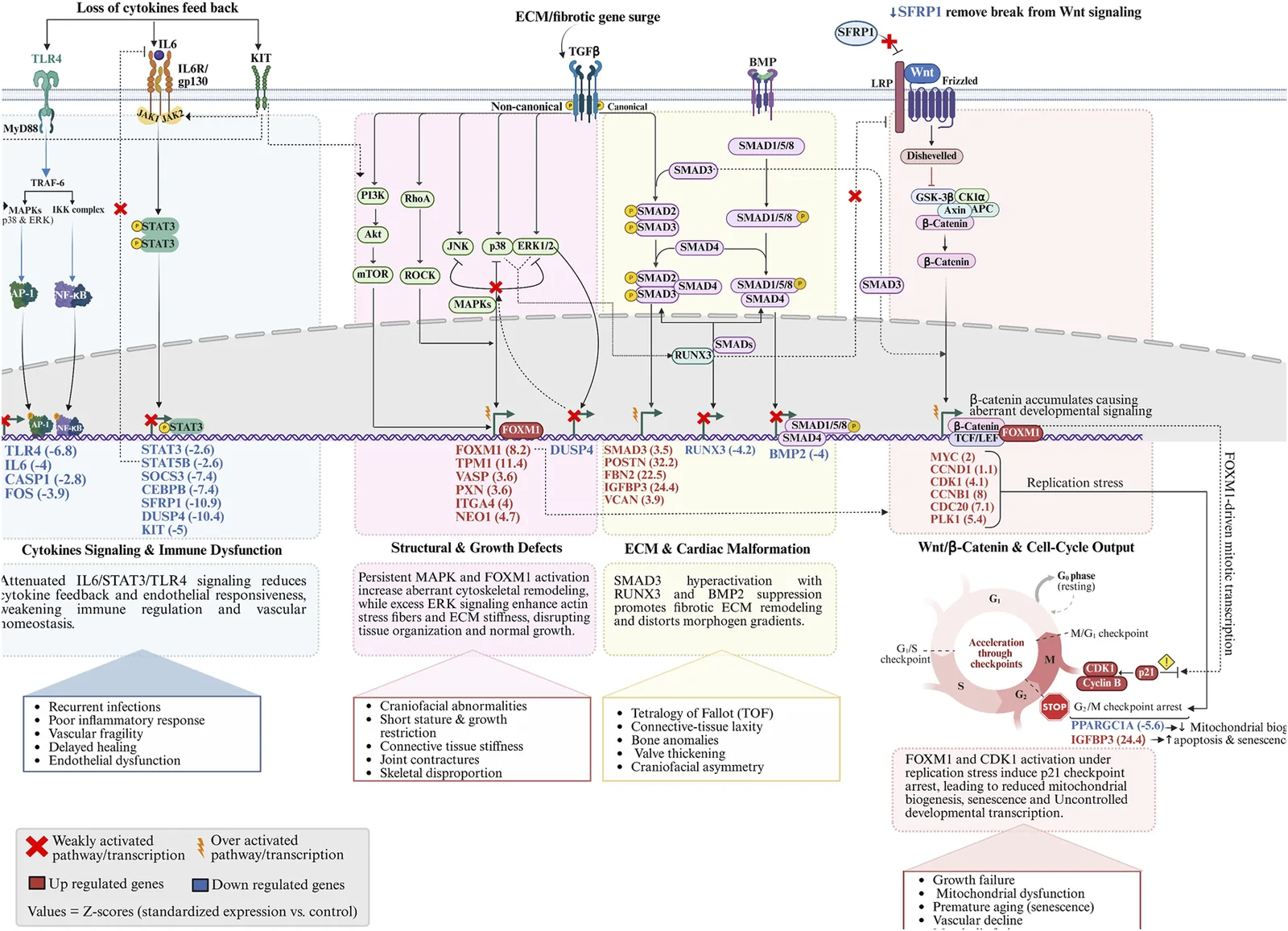
Integrated model of pathway dysregulation associated with *LARP7* deficiency. The schematic summarizes dysregulated signaling networks identified through transcriptomic, pathway enrichment, and network analyses of patient-derived fibroblasts and their potential relationships to clinical manifestations of Alazami syndrome. Reduced cytokine signaling (TLR4/IL6–STAT3 axis) is associated with impaired immune and vascular regulatory pathways, whereas MAPK–FOXM1 and Wnt/β-catenin signaling is consistent with cytoskeletal remodeling, extracellular matrix (ECM) gene expression, and cell-cycle dysregulation. Reduced BMP2 and SFRP1 expression, together with dysregulation of SMAD3 and ECM-associated genes, is consistent with altered developmental signaling and ECM remodeling. Gene-expression changes and corresponding Z-scores are derived directly from the patient-versus-control RNA-seq analysis, whereas pathway relationships and proposed biological consequences are literature-supported interpretations. Clinical manifestations shown represent phenotypes observed in the present patient and/or previously reported individuals with Alazami syndrome. These relationships are hypothesis-generating and do not establish a causal relationship between the observed transcriptomic changes and TOF. Upregulated and downregulated genes are shown in red and blue, respectively (Z-scores, patient versus control). Red “X” symbols indicate reduced pathway activity, whereas arrows denote reported or inferred signaling relationships.

**TABLE 3:** Differentially expressed genes with strong, direct evidence from human mutations or well-established developmental roles in tetralogy of Fallot (TOF).

Rank	Gene	Direction	Evidence summary^*^
1	ACTC1 (Cardiac α-actin)	Up	Human mutations cause TOF and other septal defects. Essential for sarcomere and outflow tract development. Several TOF families show ACTC1 variants
2	POSTN (Periostin)	Up	Knockout mice display outflow tract malformations and valve defects mimicking TOF. Expressed in endocardial cushions and neural crest–derived cells
3	DKK1 (Dickkopf-1)	Up	Inhibitor of Wnt/β-catenin signaling. Wnt disruption causes conotruncal malformations, including TOF, in both human and model studies
4	FBN2 (Fibrillin-2)	Up	Mutations cause congenital connective tissue syndromes with outflow tract anomalies and aortic root defects. Expressed in embryonic heart and aorta
5	CRIM1 (Cysteine-rich BMP modulator 1)	Up	Controls BMP and VEGF signaling in valve and septal morphogenesis. Mouse models show outflow tract and cushion defects resembling TOF.
6	NTN1 (Netrin-1)	Down	Guides cardiac neural crest cell migration. Loss or dysregulation leads to outflow tract septation defects — a key TOF mechanism
7	SFRP1 (Secreted Frizzled-Related Protein 1)	Down	Wnt modulator; aberrant Wnt inhibition during conotruncal development leads to TOF-like malformations

^*^ Based on human genetic studies, animal knockout phenotypes, or mechanistic developmental data.

While these observations derive from fibroblast transcriptomic analysis and do not establish causality, they identify candidate molecular processes that warrant investigation in disease-relevant models. Notably, heart-specific sensitivity to 7SK-snRNP disruption has precedent; HEXIM1 loss causes fetal cardiac hypertrophy and lethality, suggesting the developing heart is especially reliant on tight P-TEFb control (Byers et al., 2005). Recent studies also implicate *LARP7* in vascular aging and cardiac mitochondrial function, further supporting its importance in cardiovascular homeostasis (Yu et al., 2021).

From a clinical perspective, the spectrum of congenital cardiac abnormalities reported in individuals with Alazami syndrome, as summarized in Table 1, together with the TOF observed in the present patient, supports consideration of baseline cardiac evaluation, including echocardiography, in individuals with confirmed *LARP7* deficiency.

### 4.3 Study limitations

This study is limited by its single-subject design and use of a single control, which precludes biological replication and cannot fully account for inter-individual or genetic-background variability. Although an N-of-1 framework was used to address the analytical constraints of non-replicated data, the transcriptomic findings should be considered exploratory and require validation in larger *LARP7*-deficient cohorts. In addition, key differentially expressed genes identified by RNA-seq were not independently validated by qRT-PCR and should therefore be considered hypothesis-generating. An additional consideration is the use of dermal fibroblasts as the transcriptomic model. While fibroblasts provide an accessible primary patient-derived system for examining transcriptional alterations associated with *LARP7* deficiency, they do not recapitulate the cellular context of cardiac or neurodevelopmental tissues. Accordingly, the observed molecular signatures should not be interpreted as direct mechanisms underlying these phenotypes but rather as candidate processes for investigation in disease-relevant models.

Nevertheless, the convergence of differential expression, pathway enrichment, and network analyses supports the biological coherence of the identified molecular signatures. N-of-1 transcriptomic frameworks have been successfully applied in rare genetic diseases and can identify biologically coherent pathway perturbations as reflected by the convergence of our transcriptomic findings. Although no additional pathogenic or likely pathogenic variants were identified in established congenital heart disease genes, an independent genetic contribution to TOF cannot be fully excluded, as its genetic basis remains incompletely understood and not all causative genes are known. Additionally, because exome sequencing primarily detects single-nucleotide variants and small insertions/deletions, the contribution of structural or noncoding genomic variants cannot be excluded. Future studies incorporating larger Alazami syndrome cohorts and complementary functional models, including iPSC-derived cardiac cells, cardiac organoids, isogenic systems, and animal models, are needed to validate these findings and further define the role of *LARP7* in developmental and cardiac pathobiology.

## 5 Conclusion

This study provides the first transcriptome-wide characterization of genetically confirmed *LARP7* deficiency in primary human cells and potentially expands the phenotypic spectrum of Alazami syndrome to include tetralogy of Fallot. *LARP7* deficiency was associated with widespread transcriptional dysregulation, marked by activation of extracellular matrix remodeling and cell-cycle pathways alongside suppression of immune and developmental signaling programs. Integrated transcriptomic, pathway enrichment, and network analyses identified dysregulation of STAT3-and SMAD3-associated pathways together with perturbation of extracellular matrix and cytoskeletal networks, highlighting candidate molecular processes for future investigation. Collectively, these findings highlight a critical role for *LARP7* in the regulation of developmental and morphogenetic programs and provide new insights into the molecular pathogenesis of Alazami syndrome. More broadly, this work demonstrates the value of transcriptomic profiling in rare Mendelian disorders and establishes a foundation for future genotype–phenotype studies and functional investigations of *LARP7*-related disease.

## Data Availability

The datasets presented in this study can be found in online repositories. The names of the repository/repositories and accession number(s) can be found in the article/Supplementary Material.
