# Supplementary material for: A novel LARP7 variant expands the phenotypic spectrum of Alazami syndrome and reveals transcriptomic dysregulation of developmental and extracellular matrix pathways
Source: Front Cell Dev Biol. 2026 Oct 14;14:1904805. doi: 10.3389/fcell.2026.1904805 (PMC13713625; doi:10.3389/fcell.2026.1904805)
Supplement: Supplementary file 1 [file DataSheet1.pdf]

Supplementary Table S1. Variants identified in key tetralogy of Fallot/congenital heart disease-associated genes in trio-exome sequencing.

Gene	Variant (HGVS)	Consequence	DP	GQ	gnomAD exome AF	ClinVar classification
NOTCH1	NM_017617.5:c.1441+7C>T	splice region variant	86	213	0.576858	Benign
NOTCH1	NM_017617.5:c.1862G>A (NP_060087.3:p.Arg621His)	missense variant	148	48	0.00103543	Likely Benign
NOTCH1	NM_017617.5:c.2265T>C (NP_060087.3:p.Asn755=)	synonymous variant	153	48	0.400666	Benign
NOTCH1	NM_017617.5:c.2588-4G>A	splice region variant	93	48	0.475265	Benign
NOTCH1	NM_017617.5:c.312T>C (NP_060087.3:p.Asn104=)	synonymous variant	126	376	0.587414	Benign
NOTCH1	NM_017617.5:c.5094C>T (NP_060087.3:p.Asp1698=)	synonymous variant	89	48	0.449445	Benign
NOTCH1	NM_017617.5:c.6555C>T (NP_060087.3:p.Asp2185=)	synonymous variant	120	316	0.621932	Benign
NOTCH1	NM_017617.5:c.852G>A (NP_060087.3:p.Pro284=)	synonymous variant	112	48	0.135412	Benign
FLT4	NM_182925.5:c.*29G>C	3 prime UTR variant	80	48	0.000244371	Not available
FLT4	NM_182925.5:c.2670C>G (NP_891555.2:p.His890Gln)	missense variant	82	48	0.630037	Benign
FLT4	NM_182925.5:c.3198C>T (NP_891555.2:p.Pro1066=)	synonymous variant	126	47	0.265059	Benign
FLT4	NM_182925.5:c.3437G>A (NP_891555.2:p.Arg1146His)	missense variant	137	48	0.0747722	Benign
FLT4	NM_182925.5:c.445A>G (NP_891555.2:p.Asn149Asp)	missense variant	83	46	0.0737674	Benign
MYH6	NM_002471.4:c.2151C>T (NP_002462.2:p.Tyr717=)	synonymous variant	128	47	0.0242353	Benign
MYH6	NM_002471.4:c.3302T>C (NP_002462.2:p.Val1101Ala)	missense variant	94	280	0.336849	Benign
MYH6	NM_002471.4:c.3388G>A (NP_002462.2:p.Ala1130Thr)	missense variant	153	42	0.094652	Benign
MYH6	NM_002471.4:c.411G>A (NP_002462.2:p.Glu137=)	synonymous variant	126	48	0.207004	Benign
MYH6	NM_002471.4:c.427C>A (NP_002462.2:p.Arg143=)	synonymous variant	130	48	0.0954837	Likely Benign
MYH6	NM_002471.4:c.4914T>C (NP_002462.2:p.Ala1638=)	synonymous variant	82	48	0.452153	Benign
NKX2-5	NM_004387.4:c.63A>G (NP_004378.1:p.Glu21=)	synonymous variant	75	43	0.407742	Benign
TBX1	NM_080647.1:c.1189A>C (NP_542378.1:p.Asn397His)	missense variant	30	48	0.250557	Benign
TBX1	NM_080647.1:c.420T>C (NP_542378.1:p.Phe140=)	synonymous variant	130	48	0.242657	Benign
TBX1	NM_080647.1:c.933A>G (NP_542378.1:p.Ala311=)	synonymous variant	64	47	0.229858	Benign
JAG1	NM_000214.3:c.2214A>C (NP_000205.1:p.Thr738=)	synonymous variant	95	241	0.11107	Benign
JAG1	NM_000214.3:c.2612C>G (NP_000205.1:p.Pro871Arg)	missense variant	178	48	0.063421	Benign
JAG1	NM_000214.3:c.267G>A (NP_000205.1:p.Gly89=)	synonymous variant	81	48	0.0960197	Benign
JAG1	NM_000214.3:c.3417T>C (NP_000205.1:p.Tyr1139=)	synonymous variant	121	361	0.651318	Benign
JAG1	NM_000214.3:c.744A>G (NP_000205.1:p.Pro248=)	synonymous variant	124	48	0.0723235	Benign
TBX5	NM_000192.3:c.*97G>A	3 prime UTR variant	98	48	0.610751	Benign
KDR	NM_002253.4:c.*27T>C	3 prime UTR variant	77	187	0.997056	Not reported

Abbreviations: DP, read depth; GQ, genotype quality; AF, allele frequency. DP and GQ refer to individual variant positions.
