# Supplementary material for: A novel LARP7 variant expands the phenotypic spectrum of Alazami syndrome and reveals transcriptomic dysregulation of developmental and extracellular matrix pathways
Source: Front Cell Dev Biol. 2026 Oct 14;14:1904805. doi: 10.3389/fcell.2026.1904805 (PMC13713625; doi:10.3389/fcell.2026.1904805)
Supplement: Supplementary file 2 [file Table1.docx]

Supplementary Material

# Supplementary Materials Legend

**Supplementary Figure S1.** Quality control assessment of RNA-seq data for patient (RP190724; left panels) and control (RC010724; right panels). A) Raw read statistics showing proportions of clean reads, adapter contamination, low-quality reads, and ambiguous bases. B) Base composition distribution across clean reads, demonstrating balanced nucleotide frequencies and negligible N content. C) Base quality profiles across read lengths, indicating consistently high Phred scores. D) Read distribution across transcripts, showing typical 5′–3′ coverage profiles. E) Transcript coverage statistics, indicating that most transcripts achieved near-complete coverage. F) Sequencing saturation curves, demonstrating plateauing of gene identification with increasing read depth.

**Supplementary Figure S2.** Overview of transcriptomic analysis and pathway enrichment in the patient sample. A) Histogram of standardized Z-scores with empirical null distribution fitted using the locfdr approach to estimate local false discovery rates and identify DEGs. B, C, D MA plots illustrate variance stabilization and differential expression. B) Raw counts show high dispersion at low expression levels. C) Variance-stabilized (VST) values display uniform variance across the range. D) MA plot showing upregulated (red) and downregulated (blue) DEGs.(E–H) Cross-database pathway concordance. E) Sankey diagram linking GO: BP (grey) pathways with Reactome (green) and KEGG (blue) modules via shared DEGs. F–G) Jaccard similarity heatmaps showing pathway overlap. F) Jaccard similarity heatmap between Reactome and GO BP. G) Jaccard similarity heatmap between KEGG and Go BP.

**Supplementary File 1:**

File format: .xlsx

Title of data: Differentially expressed genes (DEGs) between patient and control samples, identified at |Z| ≥ 2 and locfdr ≤ 0.20.

Description of data: Significantly up- or downregulated genes (in red) are shown with Z-scores, false discovery rates (locfdr, q_BH), effect sizes (D*), and their variability (MAD_D*), along with mean expression values in controls (h_control) and patients (h_patient), and sample counts. Functional annotations summarize each gene’s biological role.

**Supplementary File 2:**

File format: .xlsx

Title of data: Enriched Reactome pathways.

Description of data: Significantly enriched Reactome pathways among DEGs, including pathway size, gene count distributions, enrichment statistics (p-value, odds ratio), BY-adjusted q-values, and direction of regulation.

**Supplementary File 3:**

File format: .xlsx

Title of data: Enriched KEGG pathways.

Description of data: Significantly enriched KEGG pathways among DEGs, including pathway size, gene count distributions, enrichment statistics (p-value, odds ratio), BY-adjusted q-values, and direction of regulation.

**Supplementary File 4:**

File format: .xlsx

Title of data: Enriched pathways by N-of-1 MixEnrich GO BP.

Description of data: Upregulated genes showed strong enrichment in mitotic and chromosome segregation processes, with odds ratios, p-values, and FDR-adjusted q-values (Benjamini–Yekutieli) reported.

**Supplementary File 5:**

File format: .xlsx

Title of data: Downregulated pathways by N-of-1 MixEnrich.

Description of data: The Table shows pathway size, number of downregulated genes in the set, number outside the set, odds ratios, p-values, and FDR-adjusted q-values (Benjamini–Yekutieli).

**Supplementary File 6:**

File format: .xlsx

Title of data: Clustered top 50 upregulated GO Biological Process pathways identified by N-of-1 MixEnrich.

Description of data: Pathways were filtered at FDR < 0.05 and subsequently grouped into non-redundant functional clusters based on semantic-similarity analysis. Shown are pathway size, fold enrichment, representative genes, and functional cluster assignments.

**Supplementary File 7:**

File format: .xlsx

Title of data: Clustered top 50 down-regulated GO Biological Process pathways identified by N-of-1 MixEnrich.

Description of data: Pathways were filtered at FDR < 0.05 and subsequently grouped into non-redundant functional clusters based on semantic-similarity analysis. Shown are pathway size, fold enrichment, representative genes, and functional cluster assignments.

**Supplementary File 8:**

File format: .xlsx

Title of data: Network-based prioritization of the top five driver genes for upregulated pathways.

Description of data: Top five driver genes per upregulated pathway based on network-based prioritization, including DEG metrics, centrality values, pathway recurrence, and DriverScore.

**Supplementary File 9:**

File format: .xlsx

Title of data: Network-based prioritization of the top five driver genes for each downregulated pathway.

Description of data: Genes were ranked using differential expression (Z, locfdr), network centrality (degree and betweenness), recurrence across pathways, and the composite DriverScore.
